# Apoptosis-mediated anticancer activity in prostate cancer cells of a chestnut honey (*Castanea sativa* L.) quinoline–pyrrolidine *gamma*-lactam alkaloid

**DOI:** 10.1007/s00726-021-02987-9

**Published:** 2021-05-04

**Authors:** Giangiacomo Beretta, Roberta Manuela Moretti, Rita Nasti, Raffaella Cincinelli, Sabrina Dallavalle, Marina Montagnani Marelli

**Affiliations:** 1 Department of Environmental Science and Policy, University of Milan, 20133 Milan, Italy; 2 Department of Pharmacological and Biomolecular Sciences, University of Milan, 20133 Milan, Italy; 3 Department of Pharmaceutical Sciences, University of Milan, 20133 Milan, Italy; 4 Department of Food, Environmental and Nutritional Sciences, University of Milan, 20133 Milan, Italy

**Keywords:** Kynurenic acid, Quinoline alkaloids, Tryptophan metabolism, Prostate cancer, Apoptosis

## Abstract

Prostate cancer (PCa) is the most common malignancy in men and represents the second leading cause of cancer deaths in Western countries. PCa is initially androgen-dependent, however, this tumor inevitably progresses as castration-resistant prostate cancer (CRPC), which represents the most aggressive phase of the pathology. In this work, in two CRPC cell lines (DU145 and PC3), we studied the in vitro inhibitory properties of the tryptophan-derived endogenous metabolite kynurenic acid (KYNA) and of the lactam form of 3–2′-pyrrilonidinyl-kynurenic acid (3-PKA-L), alkaloids usually present in combination in chestnut honey. Cytotoxicity was evaluated by 3-(4,5-dimethylthiazole-2-yl)-2,5-diphenyltetrazolium bromide (MTT) assay, cell colony formation assay, and Western blot analysis of the major mediator proteins involved in apoptotic processes. In all experiments, KYNA was scarcely or not active while 3-PKA-L showed anticancer activity in the high concentration range (0.01 mM – 1 mM) from 24 to 72 h. The results obtained showed that cell death was induced by extrinsic apoptotic pathway, by cell morphological changes and reduction of cell colonies number. These novel results represent the first promising step to the accurate description of 3-PKA-L cytotoxic effect, not observed with KYNA, paving the way to the search of new anticancer agents, as well as to the better understanding of the physiopathological role of this interesting natural product.

## Introduction

Tryptophan is an important amino acid precursor of many biological processes, and the principal route of its catabolism is represented by the kynurenine pathway.

Previous studies demonstrated that the kynurenine pathway deregulation can lead to cancer progression through alteration of the immune system anticancer activity (Adams et al. 2012; Platten et al. 2019).

KYNA, the final metabolite of the kynurenine pathway, can be produced endogenously by different types of cells (Parada-Turska et al. 2006; Kuc et al. 2006; Paluszkiewicz et al. 2009), and the initial studies regarding its biological role revealed neuroprotective and anticonvulsant properties (Scharfman et al. 2000; Erhardt et al. 2001; Chen et al. 2011).

In addition to its fundamental role as final tryptophan-derived metabolite of the kynurenine pathway, it has been recognized as a robust marker of floral origin of sweet chestnut honey (*Castanea sativa* L.). Chestnut honey, among all food types and all other honey types, shows the highest known KYNA content originating from the highly appetitive honeybee feeding sources *C. sativa* nectar and pollen.

Chestnut pollen showed a protective effect against the damage induced by carbon tetrachloride in an in vivo model of hepatic injury (Yıldız et al. 2013).

Previous studies have shown that chestnut honey components exert in vitro anti-tumoral (Seyhan et al., 2017), antioxidant (Koca et al. 2018; Küçük et al. 2007), antimicrobial (Kunčič et al. 2012), and anti-inflammatory properties (Kolayli et al. 2016).

In this foodstuff, KYNA concentration levels are at least tenfold higher comparing to any other non-carbohydrate and non-protein secondary honey component (Turski et al. 2009, 2016; Beretta et al. 2007, 2008, 2009a, b, 2012; Donarski et al. 2010; Truchado et al. 2009). For this reason, when present in the human diet, chestnut honey can be considered as the major contributor of KYNA dietary intake.

Previous studies have well defined the anti-inflammatory (Kaszaki et al. 2008), analgesic (Cosi et al. 2011), antioxidative (Lugo-Huitron et al. 2011), hepatoprotective (Marciniak et al. 2018), and antiatherogenic (Pawlak et al. 2010) activities of KYNA. However, its role in cancer development and progression is still not completely elucidated (Walczak et al. 2020).

Recent studies that analyzed the effect of KYNA on cancer cell proliferation, demonstrated an antiproliferative activity in human glioblastoma cells (Walczak et al. 2014a, b), human colon cancer cells (Walczak et al. 2014a, b), and renal cancer cells (Walczak et al. 2012).

Besides KYNA, other structurally related quinoline alkaloids have been found in chestnut honey in a wide range of concentrations (from few mg/Kg up to hundreds of mg/Kg).

**Fig. 1:**
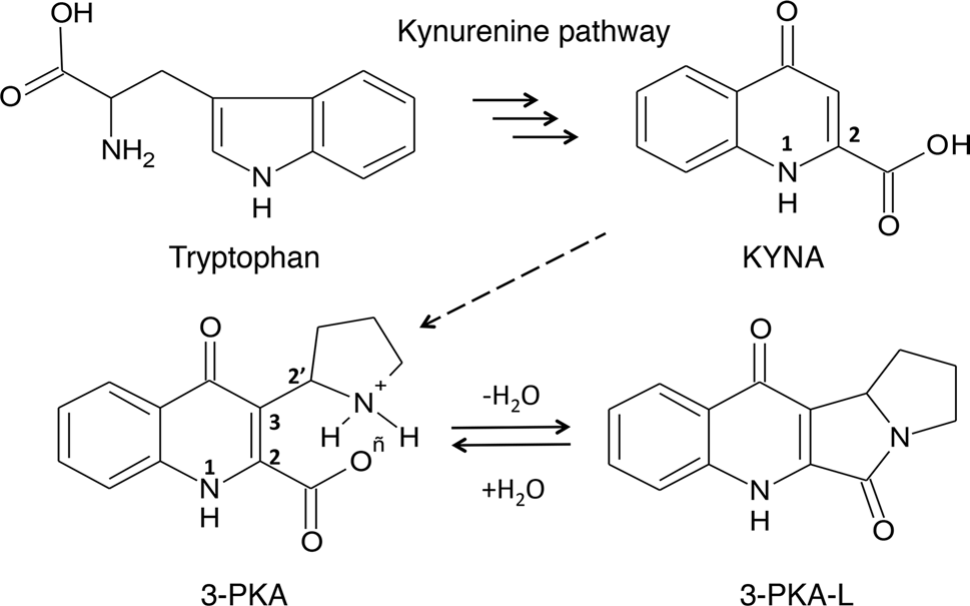
Chemical structures of tryptophan KYNA, 3-PKA, and 3-PKA-L. The dashed arrow indicates the bioconversion of KYNA to 3-PKA through an unknown pathway. The relevant atom numberings are indicated

Among these derivatives, 3-PKA represents the first example of hybrid quinoline/pyrrolidine alkaloid, which involves the activated KYNA C-3 position (Fig. 1).

Published data suggest this derivative as the major representative of a wider group of structurally related derivatives, which is sometimes able to even replace KYNA in the composition of chestnut honey (Beretta et al. 2009a, b; Truchado et al. 2009; Cho et al. 2015). To date, the factors underlying the origin of this metabolic shift at plant and/or honeybee level are unknown.

The 3-PKA open form slowly undergoes conversion to its stable tetracyclic *gamma*-lactam form (3-PKA-L) through an intramolecular dehydration mechanism. The total synthesis of 3-PKA-L has been recently published (Cincinelli et al. 2019).

The identification of 3-PKA, 3-PKA-L, and structurally related substances in chestnut honey only, with no evidence of their presence in its corresponding plant sugar/protein sources (nectar and pollen), suggests the honeybee metabolic action as the key factor in their production, de novo or possibly through the extension of the kynurenine pathway (Beretta et al. 2009a). Similarly, no information is available about the potential biochemical and biological role, as well as the bioactivity, of these derivatives.

PCa represents the second leading cause of cancer-associated mortality in Western countries with more than 1.2 million novel cases diagnosed every year worldwide (Siegel et al. 2019). Currently, surgery, radiation, and androgen-deprivation therapy (ADT) represent the treatments of choice for early-stage PCa. However, this tumor mostly progresses to a condition of CRPC and at this stage of progression the cure remains elusive (Manea et al. 2014; Testa et al. 2019). Hence, finding effective anticancer drugs endowed with low toxicity to formulate new treatment strategies is of vital importance in enhancing the survival of patients with advanced CRPC.

Thus, the aim of this study was to investigate the effects of KYNA and 3-PKA-L treatments in two CRPC cell lines (DU145 and PC3) and to evaluate whether these compounds exert cytotoxic effects triggering an apoptotic cell death process.

## Materials and methods

### Reagents

HPLC and analytical grade solvents and chemicals were purchased from Sigma (Sigma–Aldrich, St. Louis, MO, USA). Formic acid 98–100% was from Fluka (Sigma–Aldrich, St. Louis, MO, USA). Ultrapure water was obtained using a Milli-Q system (Millipore, Merck KGaA, Darmstadt, Germany). 3-PKA-L was synthesized according to the procedure previously reported (Cincinelli et al. 2018) and dissolved in dimethyl sulfoxide (DMSO). For Western blot analysis the following primary antibodies and Horseradish peroxidase-conjugate secondary antibodies were utilized: caspase 3 (9656), cleaved caspase 3 (9664), caspase 8 (4790), cleaved caspase 8 (9748), and PARP (9542) (Cell Signaling Technology Inc, Boston MA, USA). Tubulin (T6199) was from Sigma-Aldrich (St. Louis, MO, USA). The pan-caspase inhibitor carbobenzoxy-valyl-alanyl-aspartyl-[O-methyl]-fluoromethylketone (Z-VAD-FMK) was from R&D System Inc (Minneapolis, MN). The specific caspase 8 inhibitor, benzyloxycarbonyl-isoleucyl-glutamyl-threonyl-aspartic acid fluoromethyl ketone (Z-IETD-FMK) was from MedChemExpress (*MCE*®) (NJ, USA). MTT was from Sigma–Aldrich (St. Louis, MO, USA). Annexin V-FITC/PI apoptosis detection kit was from eBioscience (1030 Vienna, Austria).

### Cell cultures

The CRPC cell lines DU145 and PC3 were from American Type Culture Collection (ATCC, Manassas, VA, USA). Cells were cultured in RPMI medium supplemented with FBS (5% and 7,5%, respectively), glutamine and antibiotics. The normal human epithelial prostate RWPE-1 cell line was provided by ATCC. These cells were maintained in keratinocyte serum free medium (K-SFM9) supplemented with 0.05 mg/ml bovine pituitary extract (BPE) and 5 ng/ml epidermal growth factor (EGF). Medium was changed every 3 days. Cells were cultured in humidified atmosphere (5% CO_2_/95%, 37 °C).

### HPLC–UV/DAD analysis and 3-PKA-L stability

HPLC analyses were done as previously described with minor modifications [Beretta et al., 2009a], using a Varian LC-940 analytical/semipreparative HPLC system (Varian, Turin, Italy) equipped with binary pump, autosampler, fraction collector, a UV-DAD detector operating in the 200–400 nm range. Column for analytical separations: Kinetex™ biphenyl column, particle size 2.6 μm, pore size 100 Å, 100 × 4.6 mm column. Solvent system: 0.1% aqueous formic acid (A) and 0.1% formic acid in acetonitrile (B), flow rate = 1.6 mL/min. Gradient: 0–3 min, B = 5%; 3–15 min, from B = 5% to 60%; 15–20 min, B = 60%. Injection volume 10 µL.

3-PKA-L was analyzed in the above conditions before and after its incubation at 37 °C in cell culture medium (1 mM, neutral pH) with and without cell monolayers.

### MTT viability assay

Cells were seeded at a density of 3 × 10^4^ cells/well in 24-well plates for 24 h and then exposed to KYNA or 3PKA-L at various concentration (0.01, 0.1, 1 mM) and incubated for 24 h, 48 h, and 72 h. Control cells were treated with DMSO (vehicle) alone. Previously experiments highlighted that DMSO did not affect cell growth. After each treatment, cell viability was determined by MTT assay, as previously described (Montagnani Marelli et al. 2016). Absorbance at 550 nm was measured through an EnSpire Multimode Plate reader (PerkinElmer, Milano, Italy).

### Cell colony formation assay

Cell colony formation assay (clonogenic assay) was carried out according to Fontana et al. with minor modifications (Fontana et al. 2019). The DU145 and PC3 cells were counted using a hemocytometer (trypan blue exclusion method) and seeded in duplicates at a density of (1.0 × 10^2^ or 1.5 × 10^2^ cells/6 mL (21 cm^2^), respectively. After 48 h the cells were treated with 3-PKA-L (1 mM) for 48 h. The cells were then cultured for 12 days changing the medium twice a week. The samples were fixed with 70% methanol and stained with crystal violet 0.15%. Images of stained colonies were captured by a Nikon digital camera, and colonies were counted.

### Morphological analysis

Morphological analysis was done by optical microscopy from different fields under a Zeiss Axiovert 200 microscope with a 20 × 0.4 objective lens linked to a CoolSnap Es CCD camera (Roper Scientific-Crisel Instruments, Rome, Italy).

### Western blot assay

Western blot experiments were performed as previously described (Montagnani Marelli et al. 2016). DU145 and PC3 cells were seeded at 3 × 10^5^ cells/dish in 6-cm Petri dishes. After each treatment, adherent and floating cells were harvested and lysed in RIPA buffer (0.05 mol/L Tris.HCl pH 7.7, 0.15 mol/L NaCl, 0.8% SDS, 10 mmol/L EDTA, 100 μmol/L NaVO4, 50 mmol/L NaF, 0.3 mmol/L PMSF, 5 mmol/L iodoacetic acid) containing leupeptin (50 μg/mL), aprotinin (5 μl/mL), and pepstatin (50 μg/mL); protein preparations (35 μg) were resolved on SDS–PAGE and transferred to nitrocellulose membranes. After 1 h in blocking buffer, membranes were incubated overnight at 4 °C with the primary antibodies against caspase 8, cleaved caspase 8, caspase 3, cleaved caspase 3, PARP, and cleaved PARP. Detection was done using horseradish peroxidase-conjugated secondary antibodies and enhanced chemiluminescence (Westar Etac Ultra 2.0, XLS075,0100; Cyanagen Srl, Bologna Italy). Tubulin was utilized as a loading control.

### Annexin V/propidium iodide assay

The annexin V/propidium iodide assay was conducted according to Cristofani et al., (2018). DU145 and PC3 cells were seeded at a density of 1.5 × 10^4^ cells/dish in 6-cm Petri dishes for 24 h and then exposed to 3-PKA-L (1 mM) for 48 h. After treatment, the cells were harvested, washed with phosphate buffer solution (PBS) and resuspended in binding buffer (BB) 1X. Then cells were sequentially incubated with Annexin V-FITC and propidium iodide (PI) according to the manufacturer’s instructions. The stained cells were analyzed by flow cytometry Novocyte 3000 (Acea Bioscience, Inc) and the results analyzed by software Novo Express.

### Immunofluorescence analysis

To evaluate the release of cytochrome *c* from mitochondria, DU145 and PC3 cells were seeded at 3 × 10^4^ cell/well in 24-well plates on polylysine-coated 13-mm coverslips for 48 h before treatments. At the end of 3-PKA-L treatment, cells were incubated with MitoTracker (250 nM) for 30 min, fixed and then stained with cytochrome *c* antibody, followed by FITC-conjugated secondary antibody. Cell nuclei were stained with DAPI. Fluorescence images were captured with Zeiss Axiovert 200 microscope with a 63x/1.4 objective lens linked to a Coolsnap Es CCD camera (Roper Scientific-Crisel Instruments, Roma, Italy).

### Statistical analysis

Statistical analysis was performed with a statistic package (GraphPad Prism 5, GraphPad Software San Diego, CA, USA). Values are represented as mean ± SEM of three independent experiments. Differences between groups were analyzed using *t* test, one-way or two-way ANOVA followed by Dunnett’s or Bonferroni’s test.

## Results and discussion

### 3-PKA-L reduces cell proliferation and exerts a cytotoxic effect in prostate cancer cells

To evaluate the KYNA and 3-PKA-L anticancer potential on PCa cells, we measured DU145 and PC3 cell viability (MTT assay) after treatment with KYNA or 3-PKA-L at doses 0.01 mM, 0.1 mM, and 1 mM for 24 h, 48 h, and 72 h.

**Fig. 2:**
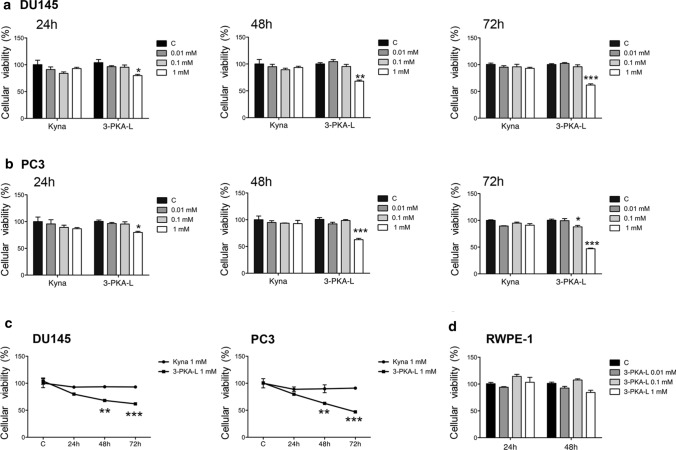
Effect of KYNA and 3PKA-L on DU145 (**a**), PC3 (**b**), and RWPE-1 cellular viability (**d**). DU145 and PC3 cells were exposed to fresh medium containing the vehicle DMSO (**c**), KYNA or 3PKA-L (0.01–1 mM) for 24, 48 and 72 h and cell viability was measured by MTT assay. Value significant in comparison to C at least with *p < 0.05, **p < 0.001, ***p < 0.0001 (one-way ANOVA with Dunnett’s post hoc test). Statistically significant differences of DU145 and PC3 cell viability between KYNA and 3PKA-L (1 mM) at 24, 48 and 72 h. This statistical analysis has been conducted with two-way ANOVA (**c**). RWPE-1 cells were exposed to fresh medium containing the vehicle DMSO (C) or to 3PKA-L (0.01–1 mM) for 24 h and 48 h and cell viability was measured by MTT assay

The results showed that 3-PKA-L was able to decrease the number of viable CRPC cells in a dose-dependent way, being significantly effective at 1 mM at all times evaluated; on the contrary, in our experimental condition, KYNA did not significantly affect the growth of CRPC cells (Fig. 2a and Fig. 2b). The latter evidence indicated that the antagonism of glutamate receptor subunits, that have been reported to modulate cancer growth, are very likely not involved in the biochemical interactions between KYNA and the CRPC cells used for testing (Luksch et al. 2011). The results reported in Fig. 2c summarize, for each cell line, the different time-dependent effect on proliferation exerted by the two molecules at the highest tested dose (1 mM). Only 3-PKA-L resulted significantly effective in reducing cell growth at 48 h and 72 h in both cell lines. On the contrary, 3-PKA-L did not significantly affect the viability of the normal prostate cells RWPE-1, both at 24 h and 48 h (Fig. 2d), demonstrating that 3-PKA-L exerts selective cytotoxic effects in CRPC cells.

**Fig. 3:**
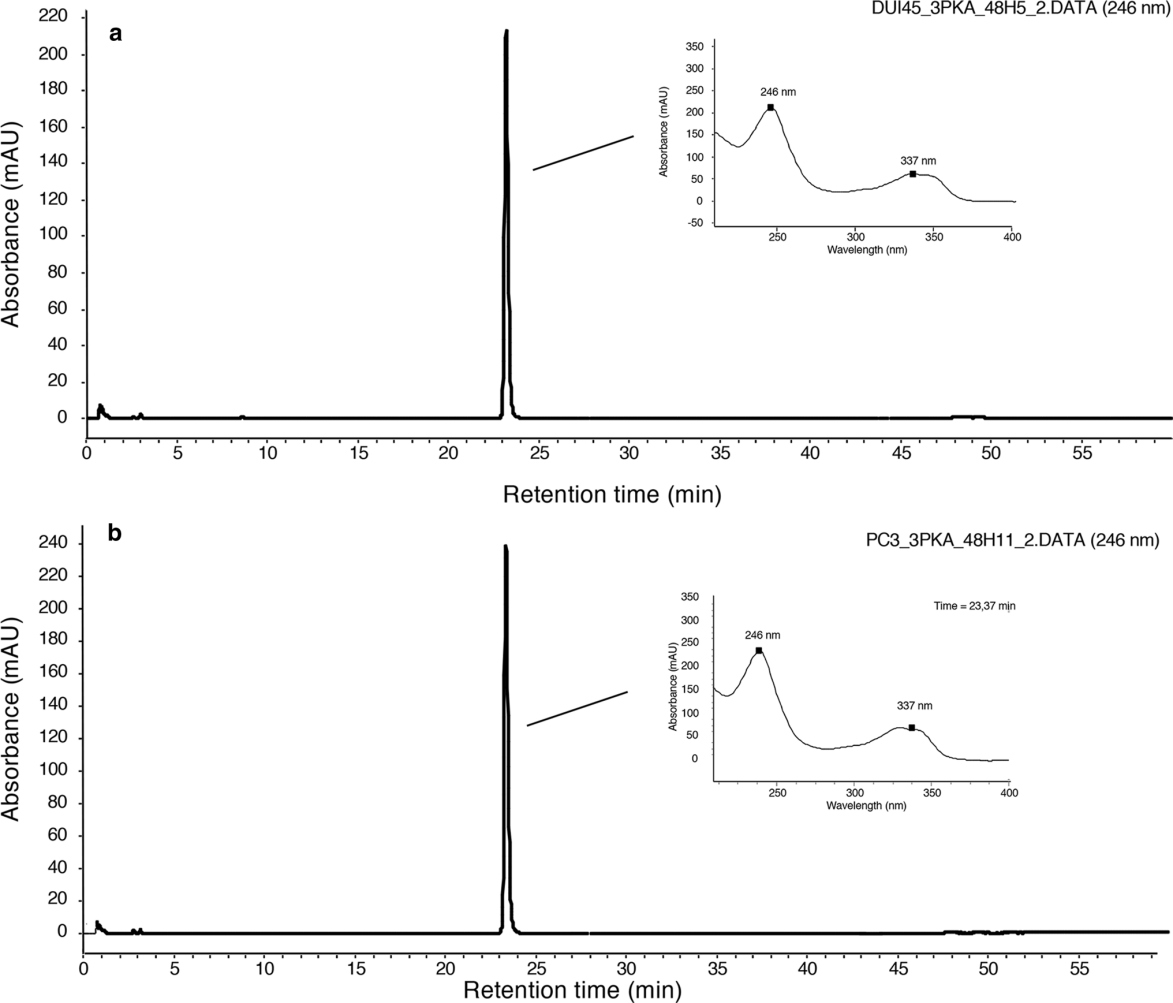
Representative HPLC–UV/DAD chromatographic profiles of (**a**) DU145 and (**b**) PC3 cell culture medium, respectively, analyzed after 48 h incubation time in the presence of 3-PKA-L. The UV spectra extracted from the main peak maximum heights are shown in the corresponding insets

Since HPLC–UV-DAD experiments indicated no significant 3-PKA-L amide bond hydrolyzation or any other medium or cell-dependent transformations, the observed antiproliferative activity was ascribed to 3-PKA-L itself in its original, unmodified chemical structure (Fig. 3).

While no literature data are available regarding the potential anticancer activity of 3-PKA-L, previous few studies reported the effect of KYNA on cancer cell proliferation.

Walczak et al. (2014a) demonstrated that KYNA at 1.3 mM concentration was able to inhibit glioblastoma T98G cells growth, to reverse the agonistic effect of glutamate on glioma cell proliferation and to enhance the antiproliferative effect of the glutamate receptor antagonists MK801 and GYKI 52,466.

Interestingly, at much lower concentrations it induced cell motility decrease, showing a synergistic effect when co-incubated with temozolomide, an anti-glioblastoma agent (Walczak et al. 2014a). The same authors showed that at higher micromolar and millimolar concentrations, which are nontoxic to normal fibroblasts, KYNA significantly inhibited human renal cancer Caki-2 cells proliferation, DNA synthesis and migration capacity. These evidences suggested that KYNA may affect cell cycle regulators and signaling pathways through simultaneous overexpression of p21 Waf1/Cip1 and inhibition of phosphorylation of Rb protein and p38 MAPK (Walczak et al. 2012).

The exposure of colon adenocarcinoma HT-29 cells to KYNA in the high-range concentration (1 mM), lead to cell growth inhibition through activation of phosphoinositide 3-kinase/Akt (PI3K/Akt) and mitogen-activated protein kinase (MAPK) signaling pathways, decreased phosphorylation of Akt, ERK 1/2, and p38 MAPK and increased expression of β-catenin expression without its nuclear translocation (Walczak et al. 2014b). The differences between our results and the above-mentioned papers about KYNA could probably be due to different types of tumor and also times of treatment. Indeed, in Caki-2 and HT-29 cells, KYNA exerts its anticancer effect at longer times (96 h) than those used by us, modifying molecular pathways related to the control of the cell cycle. These observations suggest that KYNA could exert a cytostatic but not a cytotoxic activity (Walczak et al. 2012, 2014b). Of note, in humans, the urinary levels of KYNA are reduced in PCa patients prior to radical prostatectomy, with no correlation between the concentration of the studied metabolites and the cancer grade (Gkotsos et al. 2017).

However, the present results indicate that KYNA affects cell growth only weakly and not significantly, whereas its structurally related substance 3-PKA-L has the strongest growth inhibitory capability. Therefore, 3-PKA-L was selected for the subsequent investigations and further analysis.

### Inhibition of clonogenic activity

To further test whether the growth inhibitory properties of 3-PKA-L can affect the clonogenic potential of DU145 and PC3 cells, 3-PKA-L activity was evaluated in a cell colony formation assay.

CRPC cells were treated with 3-PKA-L (1 mM, 48 h), and subsequently left to grow for 12 days in the absence of any other treatment.

After this period, the cells ability to form colonies (colonies dimensions), as well as the survival of colony-forming cells (colonies number), was analyzed.

**Fig. 4:**
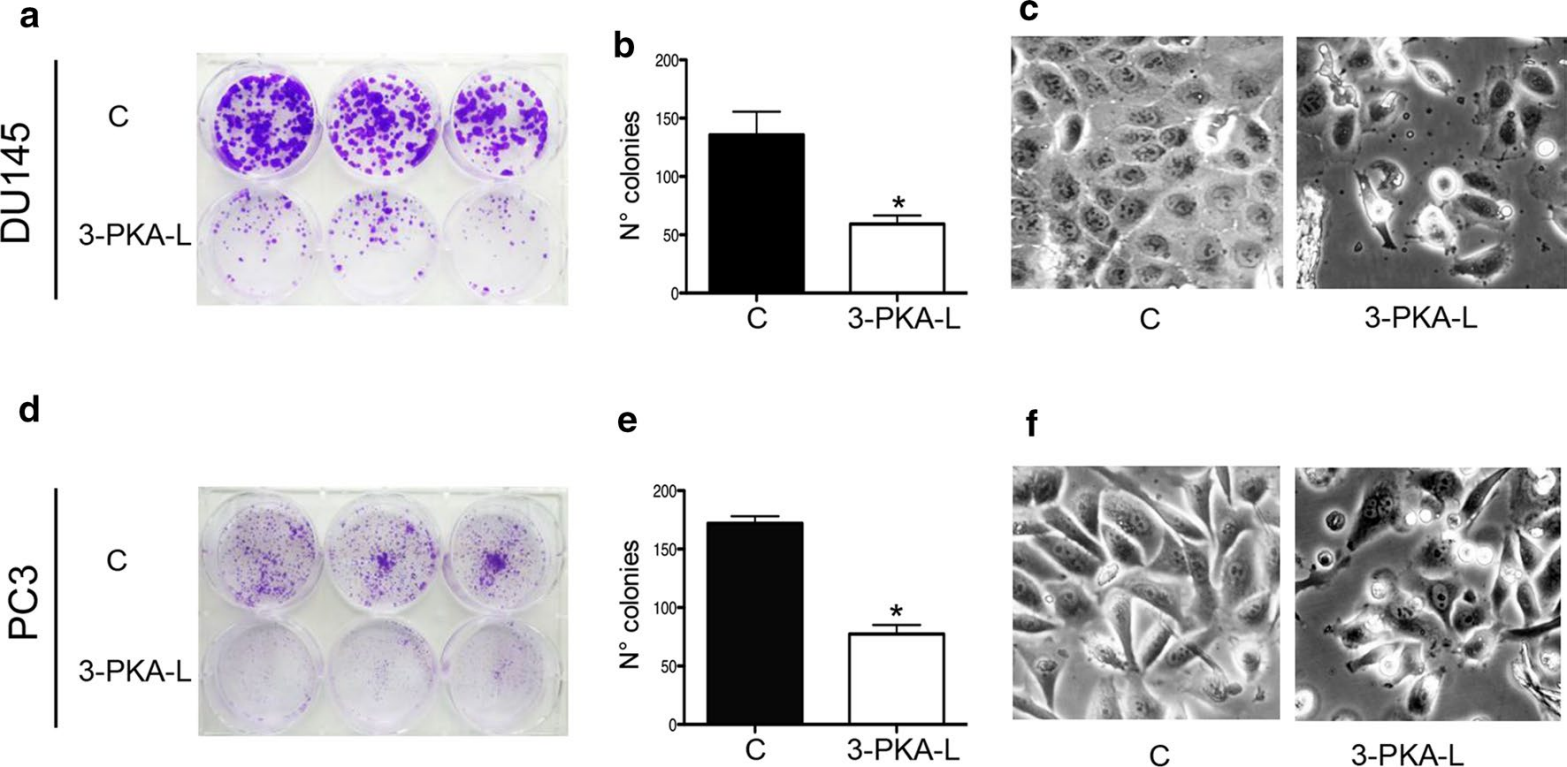
Effect of 3PKA-L on clonogenic capacity of DU145 (**a**) and PC3 (**d**) cells. Both cell lines were treated with 3PKA-L (1 mM) for 48 h, and the colony-forming ability was assessed after 12 days of culture. Cell colonies were stained with crystal violet and are represented in purple. Data were expressed as number of colonies of treated DU145 (**b**) and PC3 (**e**) cells in comparison with no treatment control cells (C). Data in (**b**) and (**e**) represent mean values ± SEM and were analyzed by t student’s test. ^*^p < 0.05 *vs* C. Morphological analysis of DU145 (**c**) and PC3 cells treated with 3-PKA-L (**f**). Morphological changes were observed after 48 h treatment with 3PKA-L (1 mM). Treated cells showed less adhesion, acquiring a spherical shape compared to control cells. This phenotype suggests a loss of cellular junctions as consequence of apoptosis induction. Cells were examined under a Zeiss Axiovert 200 microscope with a 20 × 0.4 objective lens linked to a Coolsnap Es CCD camera (Roper Scientific-Crisel Instruments)

The results showed that control cells formed colonies in a cell-type dependent pattern, with DU145 cells forming larger colonies comparing to PC3 cells. The cells exposure to 3-PKA-L (1 mM, 48 h) caused a considerable decrease in colony dimension and number. (Fig. 4a–b and Fig. 4d–e).

Moreover, as shown in Fig. 4c and f, the two cell lines underwent morphological changes in response to 3-PKA-L congruent with apoptotic events. Indeed, after treatment CRPC cells appeared more detached from the plate surface compared to control cells, acquiring a spherical shape. This phenotype suggests the involvement of a process that leads adhesion reduction and loss of cellular junctions consistent with apoptosis induction.

### 3-PKA-L triggers caspase-dependent apoptosis in CRPC cells

Apoptosis, a programmed cell death representing an important perspective on the treatment of cancer, is considered as a major cause of cell growth inhibition (Montagnani Marelli et al. 2016).

**Fig. 5:**
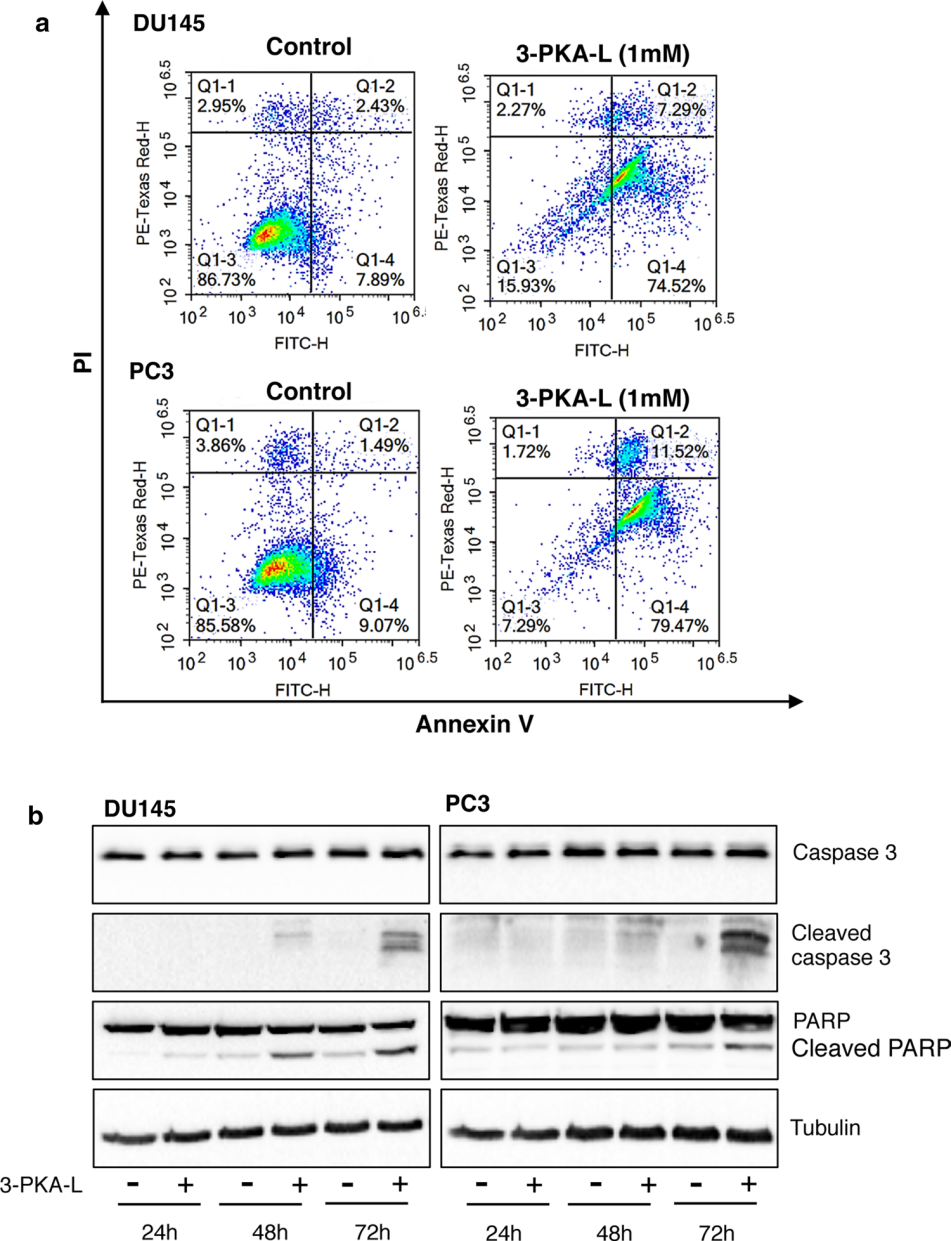
Flow cytometry analysis of CRPC cells treated with 3-PKA-L (**a**). After treatment (48), cells were stained with Annexin V/PI and analyzed by Novocyte 3000. Box Q1-1 shows necrosis, box Q1-2 shows late apoptosis, box Q1-3 shows the viable cells, and box Q1-4 shows early apoptosis. Western blot analysis of apoptosis-related proteins in CRPC cells treated with 3-PKA-L (1 mM) for 24, 48 and 72 h (**b**). Western blot was carried out on control (**C**) and 3-PKA-L-treated cellular lysates for the specified caspase or PARP proteins. Tubulin antibody was used as loading control. One representative of three different experiments performed is shown

To determine whether the cytotoxic activity of 3-PKA-L against CRPC cells resulted from apoptosis induction, Annexin V/PI staining assay and Western blot analysis of caspase 3 and its target protein PARP were carried out. The cytofluorimetric results showed that 3-PKA-L (1 mM) increased the percentage of apoptotic cells compared to the control in both cell lines (Fig. 5a). In particular, following treatment of DU145 with 3-PKA-L, 15.93% of cell remained alive, 74.52% of cells went on to early apoptosis, 7.92% of cells went into late apoptosis, and 2.27% of cells underwent necrosis. In PC3 cells, 7.29% of cells remained alive, 79.47% of cells went on to early apoptosis, 11.52% of cells went into late apoptosis, and 1.72% of cells underwent necrosis). Globally, the percentage of apoptotic DU145 and PC3 cells was 81.81% and 91%, respectively (Fig. 5a).

The Western blot results reported in Fig. 5b evidenced elevated cleavage of caspase 3 and PARP in both DU145 and PC3 cells following 3-PKA-L treatment (1 mM for 24 h, 48 h and 72 h). In particular, the levels of cleaved (active) caspase 3 were increased at 48 h and 72 h in DU145 and strongly increased after 72 h of treatment in PC3 cells.

Cleaved PARP levels increased after 48 h and 72 h in DU145 cells and after 72 h in PC3 cells (Fig. 5b). These results indicated that apoptosis, through caspase 3 activation, were involved in 3-PKA-L cytotoxicity in CRPC cells.

To further confirm the involvement of apoptosis in the antitumor activity of 3-PKA-L, CRPC cells were treated with Z-VAD-FMK, a pan-caspase inhibitor, prior to cells exposure to 3-PKA-L (1 mM, 48 h). In these conditions, the expression levels of caspase 3, PARP and their cleaved form were analyzed by Western blot. In CRPC cells, 3-PKA-L increased the expression of both active caspase 3 and cleaved PARP, as already described in Fig. 5b.

**Fig. 6:**
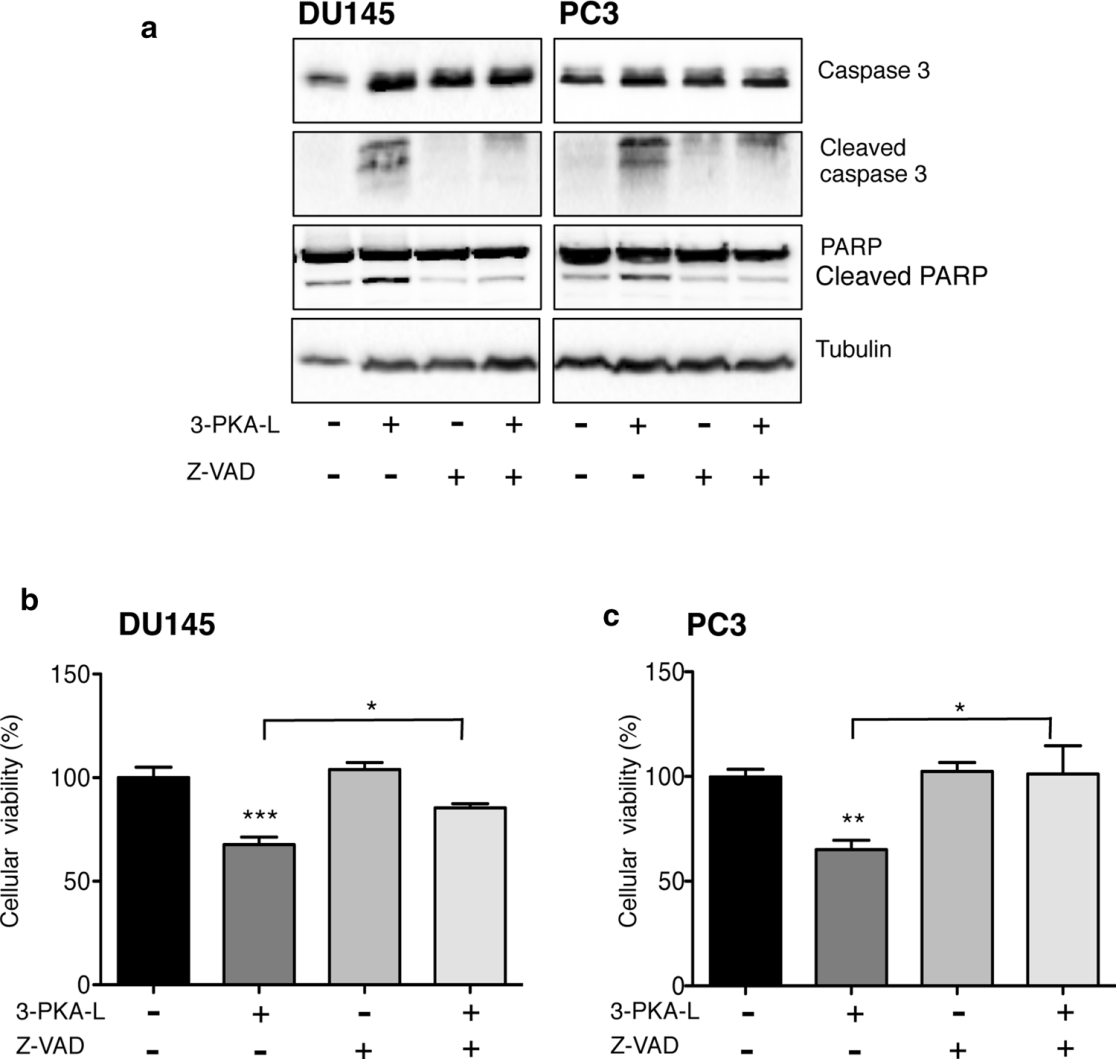
Cell viability assay to analyze the involvement of apoptosis in the anticancer activity of 3-PKA-L. DU145 and PC3 cells were pretreated with the pan-caspase inhibitor, Z-VAD-FMK (Z-VAD, 50 μM, 4 h) before the 3-PKA-L treatment (1 mM, 48 h). Cell viability and caspase 3/PARP cleavage were investigated by means of Western blot analysis (**a**) and MTT assay (**b**). In Western blot analysis, tubulin expression was used as loading control. Each experiment was repeated three times. Data in (**b**) were analyzed by Bonferroni’s test after one-way analysis of variance **p < 0.001 *vs* C; ***p < 0.0001 *vs* C; *p < 0.05

The pan-caspase inhibitor Z-VAD-FMK alone did not modify the expression of these proteins, but it significantly counteracted the 3-PKA-L effect on the expression of the cleaved form of both caspase 3 and PARP (Fig. 6a).

In CRPC cell lines, MTT assay pointed out that cell viability was significantly reduced by 3-PKA-L whereas Z-VAD-FMK alone did not affect it. Nonetheless, the treatment with this inhibitor reverted significantly (even if not completely) the anticancer effect of 3-PKA-L (Fig. 6b) confirming that the antitumor action of 3-PKA-L was related to apoptosis activation.

**Fig. 7:**
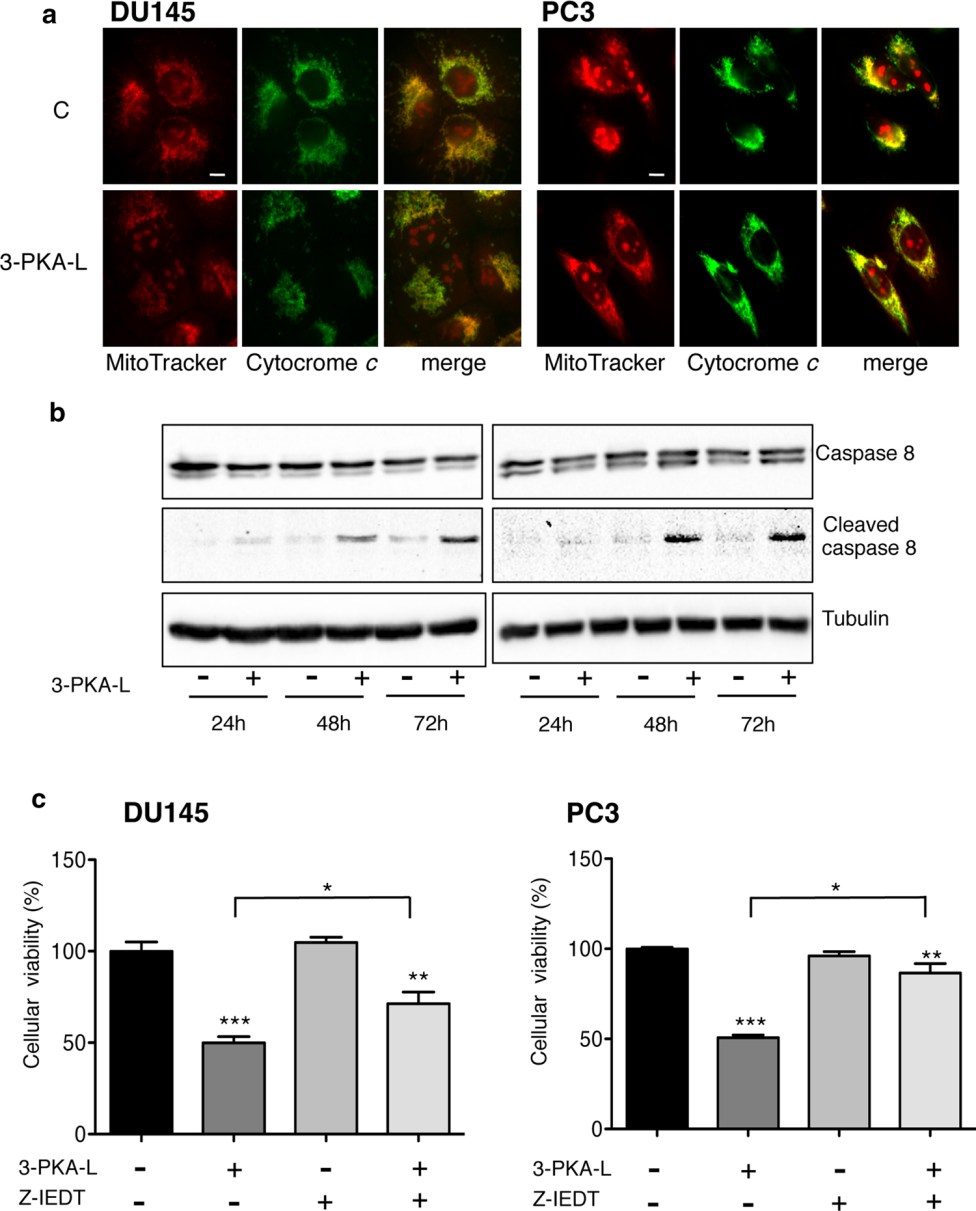
Immunofluorescence analysis to evaluate intracellular localization of cytochrome *c* (**a**). DU145 and PC3 cells, treated with 3-PKA-L (1 mM) for 48 h were then incubated for 30 min with MitoTracker, fixed and stained with cytochrome *c* antibody followed by the FITC-conjugated secondary antibody and DAPI. One representative of three different experiments performed is shown. Scale bar, 20 μm. Western blot analysis of caspase 8/cleaved caspase 8 in CRPC cells treated with 3-PKA-L (1 mM) for 24, 48 and 72 h (**b**). Western blot analysis of caspase 8 and its cleaved form was carried out on control (**C**) and 3PKA-L-treated cellular lysates. Tubulin antibody was used as loading control. One representative of three different experiments, for each of the analyses performed, is shown. Cell viability assay to analyze the involvement of extrinsic apoptosis in the anticancer activity of 3-PKA-L (**c**). DU145 and PC3 cells were pretreated with the specific caspase 8 inhibitor Z-IEDT-FMK (Z-IEDT, 50 μM, 4 h) before the 3-PKA-L treatment (1 mM, 48 h). Cell viability was investigated by means of MTT assay. Each experiment was repeated three times. Data in (**c**) were analyzed by Bonferroni’s test after one-way analysis of variance **p < 0.001 *vs* C; ***p < 0.0001 *vs* C; *p < 0.05

To evaluate whether the intrinsic apoptotic pathway was involved in the anticancer activity of 3-PKA-L, immunofluorescence studies were performed analyzing the possible release of cytochrome *c* from the mitochondria (Fig. 7a). This event was analyzed evaluating the merge between the cytochrome *c* and the MitoTracker dye accumulated in the mitochondrial membranes. The loss of this merge highlights the intrinsic apoptosis activation. The results of these experiments showed that both in DU145 and PC3 cell lines, the localization of cytochrome *c* overlapped with mitochondria in both control and treated cells. This result indicated that the intrinsic mitochondrial apoptotic pathway was not involved in the 3-PKA-L cell death activity (Fig. 7a). To assess whether the extrinsic apoptotic pathway may be involved in 3-PKA-L antitumor activity, we analyzed the expression levels of caspase 8 and its cleaved form (Fig. 7b). We demonstrated that 3-PKA-L markedly increases the expression levels of cleaved caspase 8 at 48 h and 72 h. To confirm the caspase 8 engagement, CRPC cells were treated with Z-IEDT-FMK, a specific caspase 8 inhibitor before the treatment with 3-PKA-L (1 mM 48 h). Cell viability was significantly reduced by 3-PKA-L, while Z-IEDT-FMK, given alone, did not modify cell viability; however, treatment of both cell lines with Z-IEDT-FMK significantly (even if not completely) reverted the antitumor effect of 3-PKA-L (Fig. 7c). These results demonstrate that extrinsic apoptosis pathway is involved in anticancer activity of 3-PKA-L.

This type of antiproliferative and proapoptotic activity of alkaloids belonging to diverse compound classes is not uncommonly encountered in literature (Roy et al. 2017a, b, 2018).

Typical examples of such cases are solamargine, a glycoalkaloid derived from the steroidal alkaloid solasodine, which antiproliferative action has been shown to involve the lysosomal mitochondrial cell death pathway in different types of human melanoma cancer cells (Al Sinani et al. 2016), and ukrain, a semi-synthetic substance derived from the plant species *Chelidonium majus*, able to alter the mitotic spindle microtubules dynamics in pancreatic cancer cells, leading to abnormal mitosis (Gagliano et al. 2012).

Taken collectively, our results showed for the first time that 3-PKA-L significantly induced apoptosis in DU145 and PC3 cells, suggesting extrinsic apoptosis pathways as possible molecular mechanism explaining the antiproliferative activity of this unusual KYNA structurally related compound.

Overall, the activity here demonstrated for the 3-PKA-L derivative typical of chestnut honey can also add useful information to further explain the biological actions (*e*.*g*., anti-inflammatory, anti-oxidant, etc.) described by studies from other authors for this type of monofloral honey (Seyhan et al. 2017; Koca et al. 2018; Küçük et al. 2007; Kunčič et al. 2012; Kolayli et al. 2016). Future studies to clarify whether 3-PKA-L may be involved in such activities are warranted.

## Conclusions

The current study revealed for the first time the role of 3-PKA-L in CRPC cells. This molecule, comparing to its related compound KYNA, shows a strong cytotoxic activity against human CRPC cells, providing useful and robust insight into its mechanism of action.

3-PKA-L exerts its anticancer effects promoting apoptosis of CRPC cells, in particular via the regulation of protein of extrinsic pathway. Our study revealed that 3-PKA-L possess promising anticancer properties in CRPC cells but further both in vitro and in vivo studies that deeply analyze its mechanism of action are required.

Moreover, further studies aimed at improving its pharmacological potency trough in-depth structure–activity relationships (SAR) and targeted chemical modification studies are warranted.

On the other hand, future studies on this direction may also contribute to answer the open question related to the environmental and evolutionary role of these kynurenine pathway-related metabolites in the context of the social community of honeybees, in which this metabolic pathway plays important roles. How these potential implications can translate from the insect organism to humans and the reverse, remains one exciting task to be clarified (Gonzalez 2013).
